# Seasonal Distributions and Migrations of Northwest Atlantic Swordfish: Inferences from Integration of Pop-Up Satellite Archival Tagging Studies

**DOI:** 10.1371/journal.pone.0112736

**Published:** 2014-11-17

**Authors:** John D. Neilson, Josh Loefer, Eric D. Prince, François Royer, Beatriz Calmettes, Philippe Gaspar, Rémy Lopez, Irene Andrushchenko

**Affiliations:** 1 Fisheries and Oceans Canada, 531 Brandy Cove Road, St. Andrews, New Brunswick E5B 2L9 Canada; 2 Marine Resources Research Institute, South Carolina Department of Natural Resources, PO Box 12559, Charleston, South Carolina, United States of America; 3 National Marine Fisheries Service, Southeast Fisheries Science Center, 75 Virginia Beach Drive, Miami, Florida 33149, United States of America; 4 Datasio, 22 rue Mage, 31000 Toulouse, France; 5 Collecte Localisation Satellites, Parc Technologique du Canal, 8–10 rue Hèrmes, 31526 Ramonville, France; University of Vigo, Spain

## Abstract

Data sets from three laboratories conducting studies of movements and migrations of Atlantic swordfish (*Xiphias gladius*) using pop-up satellite archival tags were pooled, and processed using a common methodology. From 78 available deployments, 38 were selected for detailed examination based on deployment duration. The points of deployment ranged from southern Newfoundland to the Straits of Florida. The aggregate data comprise the most comprehensive information describing migrations of swordfish in the Atlantic. Challenges in using data from different tag manufacturers are discussed. The relative utility of geolocations obtained with light is compared with results derived from temperature information for this deep-diving species. The results show that fish tagged off North America remain in the western Atlantic throughout their deployments. This is inconsistent with the model of stock structure used in assessments conducted by the International Commission for the Conservation of Atlantic Tunas, which assumes that fish mix freely throughout the North Atlantic.

## Introduction

Compared with other large pelagic species such as bluefin tuna (*Thunnus thynnus*, Scombridae), the horizontal movement and migrations of Atlantic swordfish (*Xiphias gladius*, Xiphiidae) are poorly described. A recent (2012) search of the literature revealed only one paper whose title included “Atlantic swordfish movements” [1], and we are aware of two further relevant contributions [2], [3], comprising a total of three papers in the primary literature as of that year. One of the three papers, [1], focussed largely upon vertical movements rather than horizontal migrations and distribution. This contrasts with the state of knowledge for bluefin tuna, where stock structure investigations using satellite tagging approaches is a particularly active field of investigation, with an analogous literature search revealing 24 published works.

Early studies using conventional “spaghetti” type tags indicated that while swordfish are capable of long distance migration, there were relatively few instances of trans-Atlantic movements (see summary in [4]). The results from those studies appear inconsistent with the stock model used by the organization responsible for management of the species (International Commission for the Conservation of Atlantic Tunas, ICCAT), who defined a management unit for stock assessment purposes of the entire North Atlantic, north of 5° N latitude. However, the absence of such trans-Atlantic movements could be related to limitations associated with conventional tagging that include low and variable rates of detection and return [5], and the often non-uniform distribution of effort that can complicate inferences of migration [6]. The development of pop-up satellite archival tags (PSATs) for the study of fish migrations has provided investigators with a tool that does not rely on the recapture and return of the tag by fishermen, thereby addressing some of the concerns surrounding the use of conventional tagging information for stock structure investigations.

A recent study [3] of swordfish migrations using the new technology found that swordfish tagged off Georges Bank in the northwest Atlantic occupy temperate waters north of 40° N latitude from early June through October, before commencing southward migration to the Caribbean Sea. The fish reach the Caribbean region by early January, where they reside until April, when the northward migration of the fish begins. While the tagged fish remained within the southern boundary of the management unit, no movement into the northeast Atlantic was found. The authors suggested this was inconsistent with the current management unit assumption.

The authors, however, acknowledged that the scope of their investigation reported in [3] was limited in several regards. It included a relatively small sample size (*n* = 10), and only one area of deployment (Georges Bank in the northwest Atlantic). Furthermore, there is the possibility that swordfish aggregations elsewhere in the western North Atlantic show greater affinities with those in the eastern North Atlantic. Another limitation of the conclusions reported in [3] was that the fish tagged were predominately large, mature individuals. This could be problematic, if mixing of the population occurs at younger stages of the life history. For example, long distance movement and migrations of Pacific halibut (*Hippoglossus stenolepis*) are thought to occur at relatively young ages [7].

In this contribution, we address the concerns of the earlier analyses by pooling results from three laboratories conducting PSAT studies of Atlantic swordfish, who have made deployments ranging from the Grand Banks of Newfoundland to waters off Florida. While taking a pooled approach provided a large number of tagged individuals for the analyses compared with other studies involving pop-up archival tags, it also provided a greater range of sizes of individuals, as well as diverse deployment locations.

Combining information from three agencies conducting PSAT-based investigations of swordfish movement has allowed us to present the most comprehensive synthesis of swordfish movements and seasonal distributions in the Northwest Atlantic produced to date. In addition, we access independent data sources such as fishery logbook information and conventional mark-recapture data to corroborate the inferences we draw from the PSAT data.

While integrating results from different investigations offers significant advantages, it also provides certain analytical challenges. For example, datasets collected from PSATs provided by different manufacturers follow different formats. Ambient variables such as pressure, temperature and light are also collected at different time resolutions, and further aggregated or binned following different strategies. High-level records include binned histograms, time series, temperature or light profiles that can be difficult to combine. The geolocation process can also differ depending on the manufacturer, based on light level fitting (e.g. Wildlife Computers), or solar event detection (e.g. Microwave Telemetry). This has implications on the error structure of light-based geolocations, and further warrants the need for filtering and smoothing to limit observation error. Several filtering methods have been published in the literature, allowing investigators to extract meaningful patterns from tagging datasets and limit the influence of the geolocation process (see, for examples [8]–[11]). These rely on filtering and smoothing observations using linear and non-linear Kalman Filter variants. Unknown parameters can be estimated via likelihood maximization, with results taking the form of a most probable track and its posterior variance estimates. For sparse datasets with complex, arbitrary constraints such as bathymetry and coastlines, an improved method was proposed in [12], using Hidden Markov Models (HMM) applied to a discretized spatial representation of the space. This method proved particularly efficient and robust when applied to tide-based geolocation of bottom fish. In this paper, we introduce an extension of this HMM-based method, using a more accurate movement model explicitly accounting for constraints and boundaries. In consequence, a secondary objective of this contribution is to describe how we integrated these diverse data.

## Methods

### PSAT Deployments

Field permits for the PSAT tagging operations were issued by Canada Department of Fisheries and Oceans and the US National Marine Fisheries Service for their respective national waters.

Three laboratories (Southeast Fisheries Science Center (Miami), South Carolina Department of Natural Resources (Charleston), and Department of Fisheries and Oceans Canada (St. Andrews)) pooled their PSAT data for the purposes of describing the movement of swordfish off the east coast of North America. All data were obtained between 2002 and 2009.

The Canadian PSATs were Wildlife Computers Mark 10 tags, deployed during between 2005–2009 inclusive. Satellite tags were attached to a modified harpoon pole following methods described in [13]. The harpoon pole was approximately 5 m long and constructed of aluminum. The distal end was modified to accept a stainless dart needle and a cushioned cradle to hold the tag. The tag was then attached to the pole using elastic bands with “breakaway” knots so that it would release when the fish was tagged. An experienced harpoon fishermen was employed to conduct the tagging, using a vessel specialized for this type of fishery [3].

The releases off Florida and Northeastern Bahamas were made by the National Marine Fisheries Service, Southeast Fisheries Science Center of the United States. Most of the PSATs were Wildlife Computers PAT4 or Mark 10 units. The PSATs were deployed off commercial pelagic longline vessels, as well as recreational vessels. Commercial buoy gear fishing vessels were also occasionally used as PSAT deployment platforms (hand-lines attached to free-floating buoys, which contain no more than two hooks per handline). Bait was squid *Illex* sp., small “tinker” mackerel *Scomber* sp., and little tunny *Euthynnus alletteratus*. Hooks used during recreational rod-and-reel fishing were size 11/0 J-style hooks, while sizes 14/0 and 16/0 non-offset circle hooks were used during the commercial longline and buoy gear operations. All PSAT tags were attached externally using a 181 kg monofilament tether and a double barb medical grade nylon anchor placed in the dorsal musculature as previously described in [14] and [15]. Some of the recent deployments used the same anchor equipped with elongated nylon toggles [1]. A 2.44 m long tagging pole was used to place the tag in the dorsal musculature, about 4–5 cm below the dorsal midline to a depth just short of exiting the opposite side of the fish, near the first dorsal fin [15], [16].

The PSATs deployed off of the Carolinas were manufactured by Microwave Telemetry Inc. (Columbia, MD, USA) and were attached to swordfish captured on pelagic longline gear deployed from the R/V Palmetto. Tags (Model PTT-100) were attached by harpooning a 6.25 cm titanium M-type dart anchor into the dorsal musculature approximately 5 cm below the midline of the base of the dorsal fin. The anchors were inserted using a 20 cm titanium tagging needle protected by a 6 cm diameter rubber stopper limiting penetration depth to 12 cm. Tags were tethered to the anchors with 30 cm of 1.66 mm diameter fluorocarbon monofilament (100 kg tensile strength). Monofilament was attached at either end with stainless steel crimps covered with adhesive-lined polyolefin heat shrink tubing. Fish were not removed from the water during tagging, and were released by removing the hook (when possible) or cutting the hook leader within a few cm of the hook.

**Figure 1:**
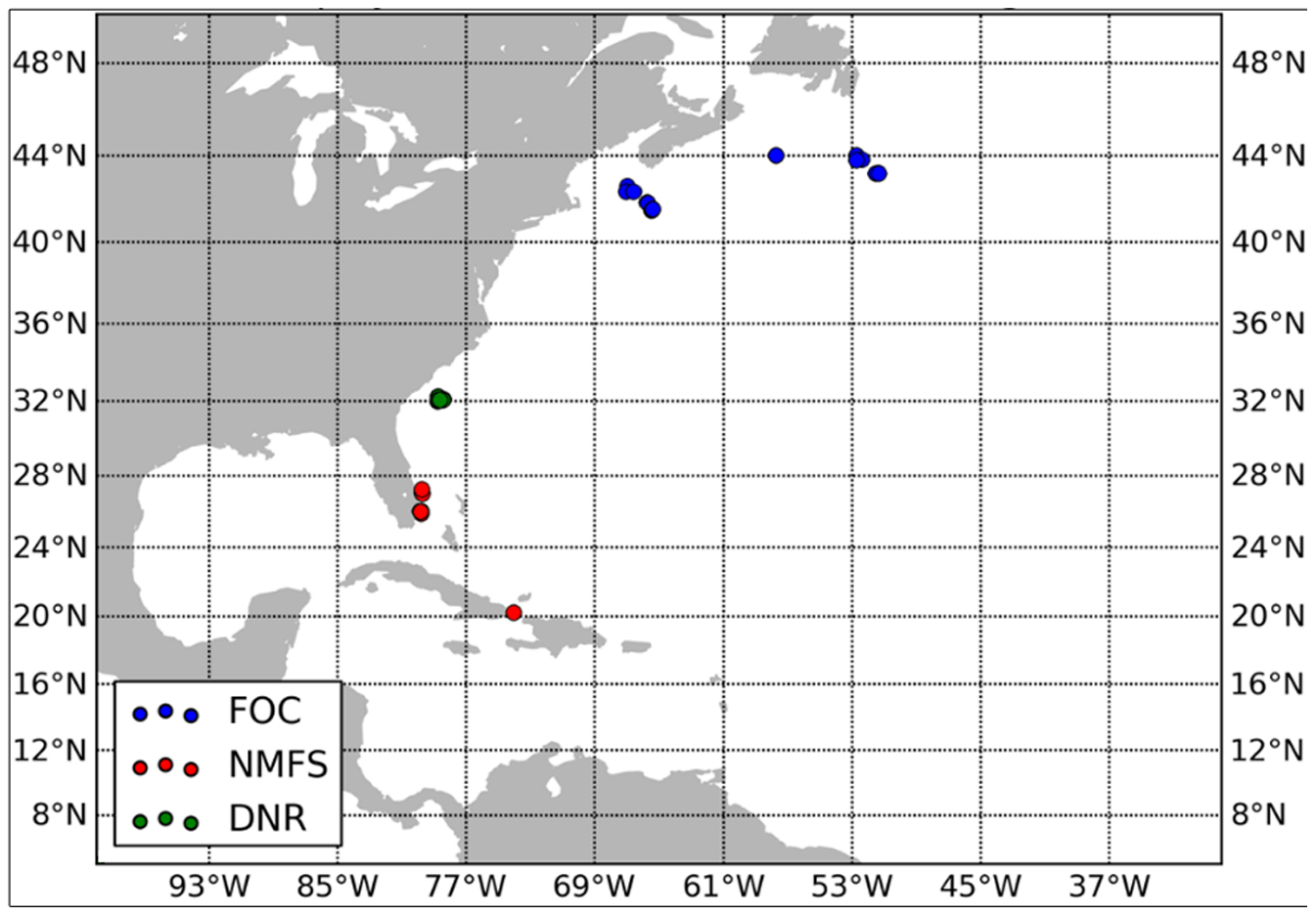
Location of releases of Atlantic swordfish marked with pop-up satellite archival tags by the National Marine Fisheries Service (NMFS), South Carolina Department of Natural Resources (DNR) and Fisheries and Oceans Canada (DFO).

The areas where deployments were made by the three laboratories are shown on Fig. 1.

**Figure 2:**
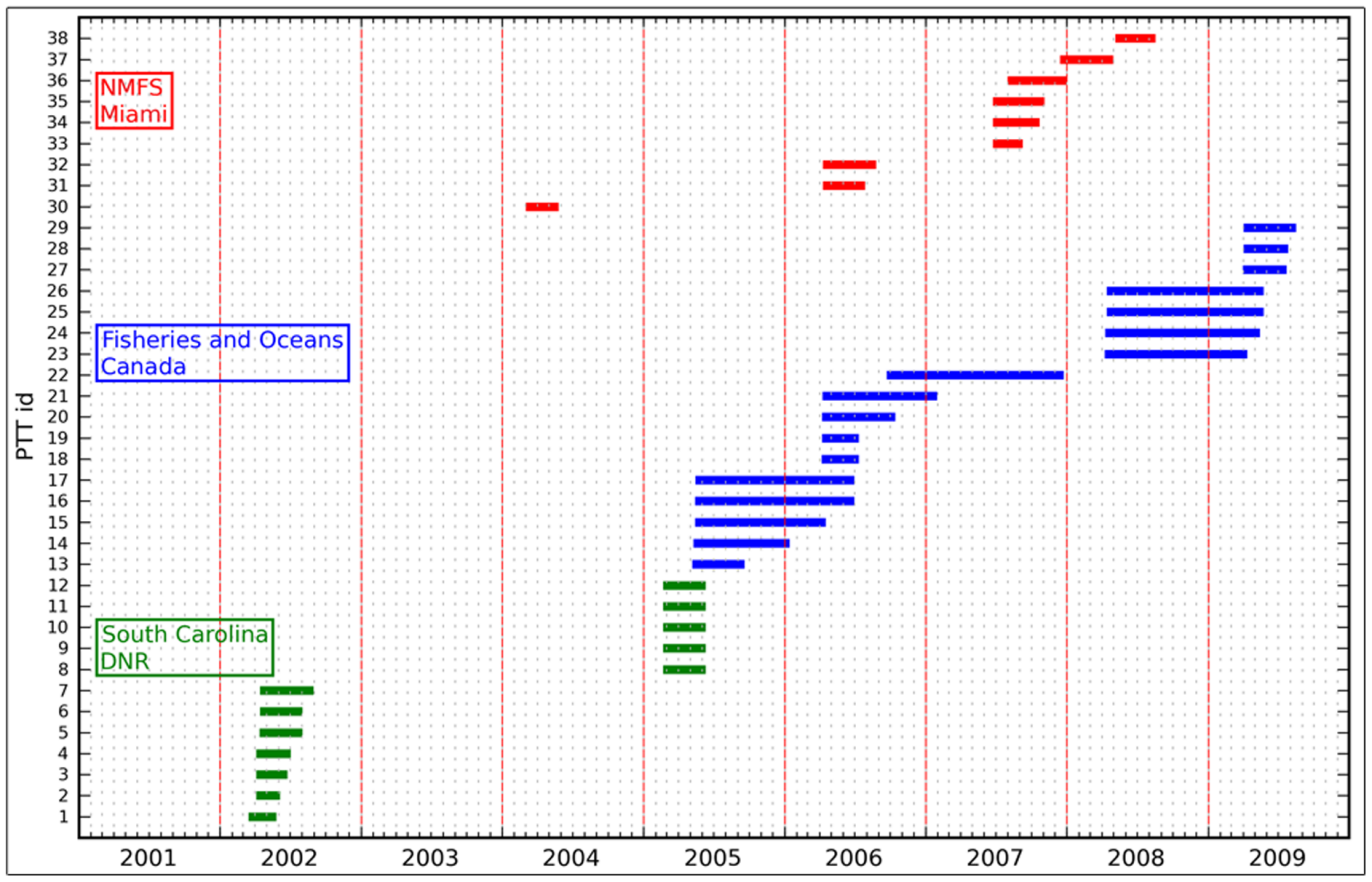
The temporal distribution of the releases of pop-up satellite archival tags on North Atlantic swordfish made by the National Marine Fisheries Service (NMFS), South Carolina Department of Natural Resources (DNR) and Fisheries and Oceans Canada (DFO). The length of the horizontal bar reflects the duration of the deployment.

From an initial set of 78 deployed PSATs, we selected 54 individuals based on a minimum deployment length of 30 days. Unfortunately, sixteen of the selected tag returns contained corrupted data due to either Argos-transmission problems, faulty sensors (light, pressure or depth), or erroneous recordings of the sensor measurements. This further reduced the number of releases finally used in the analyses to 38. A summary of the temporal distribution of PSAT deployments by the three laboratories is provided in Fig. 2. The intended deployment lengths varied, according to differing study objectives of the three agencies. Realized deployment durations were variable in all cases, and averaged 99, 83 and 249 d for the National Marine Fisheries Service (NMFS), South Carolina Department of Natural Resources (SCDNR) and Fisheries and Oceans Canada (DFO) respectively (see summary statistics concerning deployment durations, Table 1).

**Table 1:** Summary of deployments (days) by the three agencies contributing data.

Deployment Statistics by Laboratory					
	N	Minimum	Maximum	Mean	Std. Deviation
SCDNR	12	31	123	82.6	27.6
DFO	17	77	411	248.8	135.4
NMFS	9	57	135	99.2	26.7

The agencies are National Marine Fisheries Service (NMFS), South Carolina Department of Natural Resources (SCDNR) and Fisheries and Oceans Canada (DFO).

Lengths or weights were estimated at the time of release, and where weights were supplied, they were converted to lower jaw fork length using conversion factors published by ICCAT. The length of fish released varied among the three labs. The SCDNR releases were, on average, the smallest swordfish tagged: 118 cm lower jaw-fork length (LJFL) (range 81–152 cm, standard deviation 19.6 cm). The NMFS releases were on average, 155 cm LJFL (range 109–280 cm, standard deviation 53.0 cm). The DFO releases were largest, on average (mean 204 cm, LJFL range 124–263 cm, standard deviation 40.5 cm).

### Data Selection and Preprocessing

Light-based geolocation exploits sunrise and sunset times. They are linked to the animal's longitude and latitude by the following set of equations:
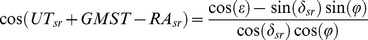


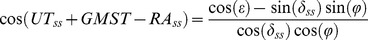
where 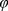 is the animal's latitude, and 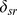, 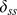 and 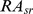, 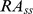 are the solar declination and right angle at sunrise and sunset. 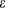 is the (predefined) sun's altitude at twilight. One can then solve for the universal time UT of sunrise and sunset, given the mean Greenwich sidereal time (GMST) which is computed from the animal's longitude λ (see [17] for a complete description of the procedure).

Methods for estimating location differ between Microwave Telemetry's PTT100 and Wildlife Computers' MK10-PAT. To derive location estimates, Microwave Telemetry's PTT100 relies on a proprietary fuzzy logic algorithm to extract timings of sunrise and sunset. Wildlife Computers' MK10-PAT transmits a subset of light levels near sunrise and sunset, to which a sun elevation model is later fitted. Subject to different noise processes and uncertainty sources, both approaches yield location estimates of similar accuracy, and are sensitive to the vertical/depth behavior of the animal. In particular, deep or repeated dives at critical times (near sunrise and sunset) yield noisy location estimates, particularly in latitude. Longitude estimates are less affected [11].

**Figure 3:**
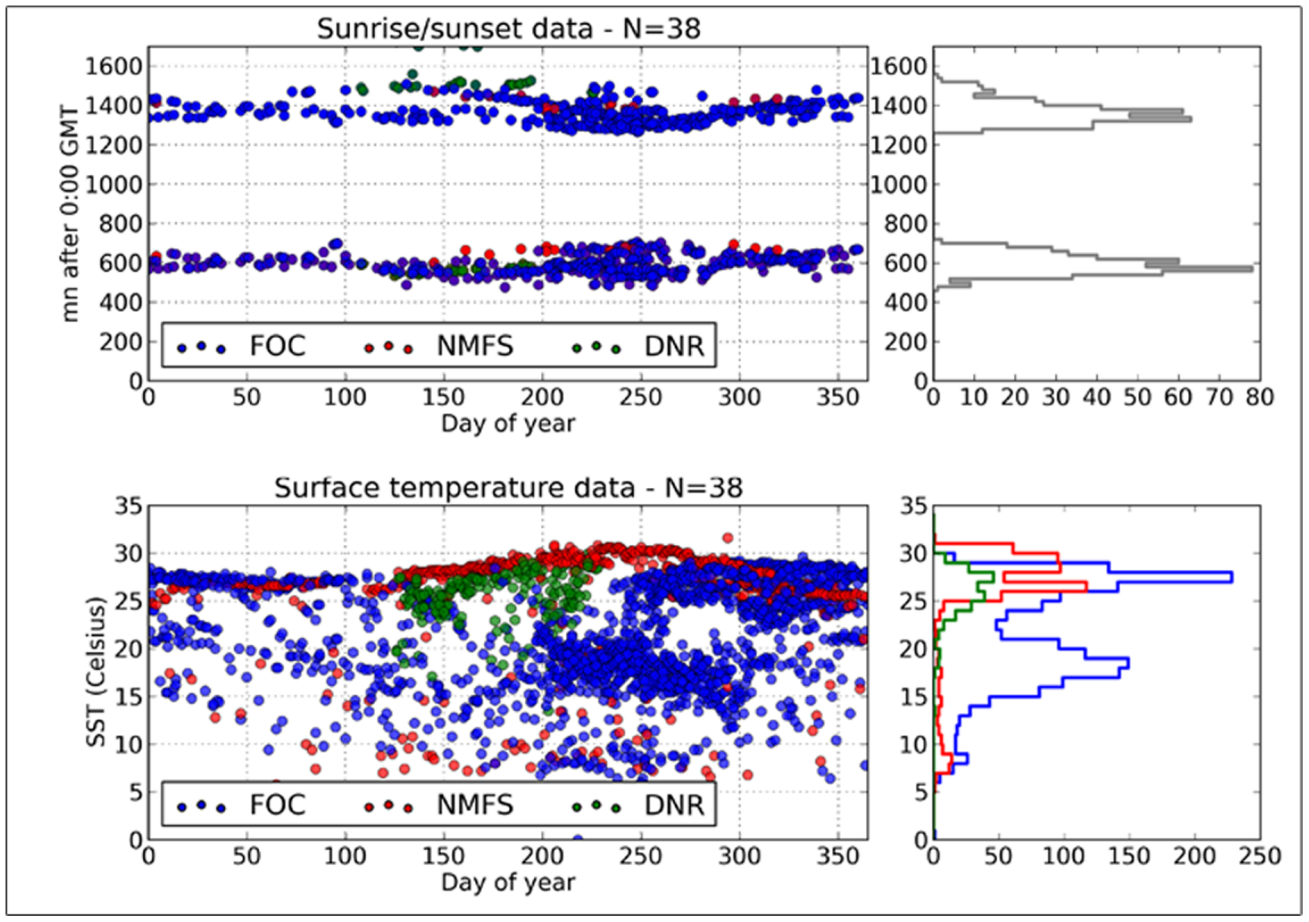
Edited sets of sunrise and sunset times from pop-up satellite archival tags deployed on North Atlantic swordfish deployed by the National Marine Fisheries Service (NMFS), South Carolina Department of Natural Resources (DNR) and Fisheries and Oceans Canada (DFO) (top panel) and SST recorded by the same tags (bottom panel). The corresponding frequency distributions of sunrise/sunset times and SST are shown on the right.

To reduce noise, sunrise and sunset times were cleaned using a Set Theoretic or Set membership approach [18]–[20] prior to applying the location processing. This method builds a daily set containing only feasible values of longitude and latitude. Starting from the deployment and pop-up locations of the tag as initial feasible values, the set is extended using forward propagation techniques, considering the maximum sustainable animal's speed, the land masks, the measured SST and the maximum measured depth. The resulting feasible set is then used to compute the likelihood of the sunrise/sunset times and to discard erroneous data. The timings of sunrise and sunset retained for the processing are shown in Fig. 3. Daily SST estimates are computed as the median temperature measured between the surface and a depth of 15 m, when the fish was present in that water layer. The median is computed from temperature-at-depth profiles for MK10 tags, and from the temperature-depth time series for PTT-100 tags. The estimated SSTs are also shown in Fig. 3 (mean SST  = 22.6°C±5.8°C). A time series of daily (SST, sunrise time, sunset time) measurements is then formed for each tag.

Unfortunately, most of these daily records were empty. Besides occasional data acquisition problems, the lack of data was essentially due to the fact that the tagged individuals spend most of the time at considerable depths (typically between 100 and 200 m), where light was so dim that measurements do not allow detection of sunrise/sunset events. The surface, or near surface, temperature measurements are also sparse as the tagged fish rarely visit the upper meters of the water column. Of a total of 6228 daily records (for 38 individuals) only 436 (7%) contain sunrise and/or sunset time measurements and 2553 (41%) contain SST data. Thus, Atlantic swordfish present a situation where positioning is essentially constrained by sparse SST measurements, only occasionally complemented by sunrise/sunset times. Under such circumstances, the determination of fish trajectories (i.e. the estimation of series of pointwise positions) cannot be reliably estimated. Instead, we implemented a grid filter approach providing estimates of the probability distribution of the successive fish positions instead of pointwise locations.

### Location Inference via Grid Filtering

Hidden Markov Models (HMM), also known as Grid Filters in the geolocation literature, rely on discretization of the state-space to infer the probability density (or the Utilization Distribution, UD) of the (hidden) location, given a sequence of observations, in this case SST and sunrise/sunset times measurements. The approach was first introduced in the field of fish tracking using depth measurements as the sole positioning information [12]. At each sampling time, the HMM method performs a position prediction step by numerically solving the advection-diffusion equation for the two-dimensional probability of animal's presence. A position update step is then performed to combine the predicted probability density with fish depth data to produce the posterior distribution of the animal. At the end of this forward filtering pass, the HMM method finally refines the filtered density estimates with a backward smoothing pass. The reader is referred to [12] for a more detailed presentation of the method.

In this study, we implement a somewhat modified version of the positioning method described in [12]. In the prediction step of the filter, the advection-diffusion equation for the probability of animal's presence is numerically solved imposing a zero-flux boundary condition both at the limits of the domain and along the coastlines. This naturally prohibits movement onto land, without leaking any probability mass. The complex boundaries of the solution domain are given by a detailed land mask at the model resolution (grid size  = 9 km). This was not achieved in [12] where the authors assumed that the domain in which the equation was solved was large enough to neglect the probability that the fish crosses the boundary. The position update step of the filter is then performed as in [12], but using SST, sunrise and sunset time instead of fish depth to produce the posterior distribution of the individual. SST and sunrise/sunset time observations are modeled as Gaussian random variables where the mean of the associated distribution is the observation value and the standard deviation is set to 1°C for SST and 20 min for sunrise and sunset time. The assumed error on SST takes into account the usual measurement error of the temperature sensors plus a sampling error due to the scarcity of temperature measurement in the upper 15 meters of water. The relatively large (20 min) error used for sunrise/sunset times is justified by the fact that their estimation suffers from the low signal to noise ratio affecting the light measurements obtained at large average depths. Note, however, that the UDs presented here are relatively insensitive to the assumed sunrise/sunset time estimation error, largely because sunrise/sunset time measurements are few and can thus have only a limited impact on the utilization distributions' estimation.

Finally, we performed a smoothing pass that slightly differs from [12] as we use the two-filter smoother formula described in [21]. It combines the previously computed filtered densities together with predicted densities given by the same filter propagating backward in time. While the forward filter is initialized with the deployment position, the backward filter is initialised with the pop-up location. This allows us to take full advantage of the accurate information provided by the pop-up position.

### Presentation of Results and Comparison with Independent Data Sets

On the assumption that the pattern of movement and migrations might differ between immature and mature fish, as observed for other teleosts [7], [22], we identified immature individuals as those less than 179 cm lower jaw fork length, and mature individuals were greater than or equal to 179 cm, following the ICCAT convention for this stock [23]. Results are presented separately for the two size-classes of swordfish.

Finally, we compared swordfish habitat use as inferred from the PSAT data to the distribution of conventionally-marked swordfish released and recaptured throughout the Atlantic since 1940. The conventional tagging database is maintained on behalf of participating countries by the International Commission for the Conservation of Atlantic Tunas (http://www.iccat.int/en/accessingdb.htm). We considered that the locations of tagging deployments and recaptures in the database provided an independent indication of the occurrence of swordfish that could be compared with the PSAT inferences.

## Results

PSAT-tagged swordfish provided relatively few estimates of sunrise and sunset (Fig. 3) that were suitable for inferences of geolocation. The data supplied by DFO was the most complete in terms of providing year-round information, consistent with the longer periods of deployment associated with that program. The combined dataset had considerably more information on SST compared with sunrise and sunset (Fig. 3). Swordfish in the northwest Atlantic experience considerable variation in sea surface temperatures concurrently, ranging from about 5 to 30°C. The data associated with the DFO program also illustrate a bimodality of temperature frequency distributions (Fig. 3), which can be seen to be linked to seasonal migrations which will be described subsequently.

**Figure 4:**
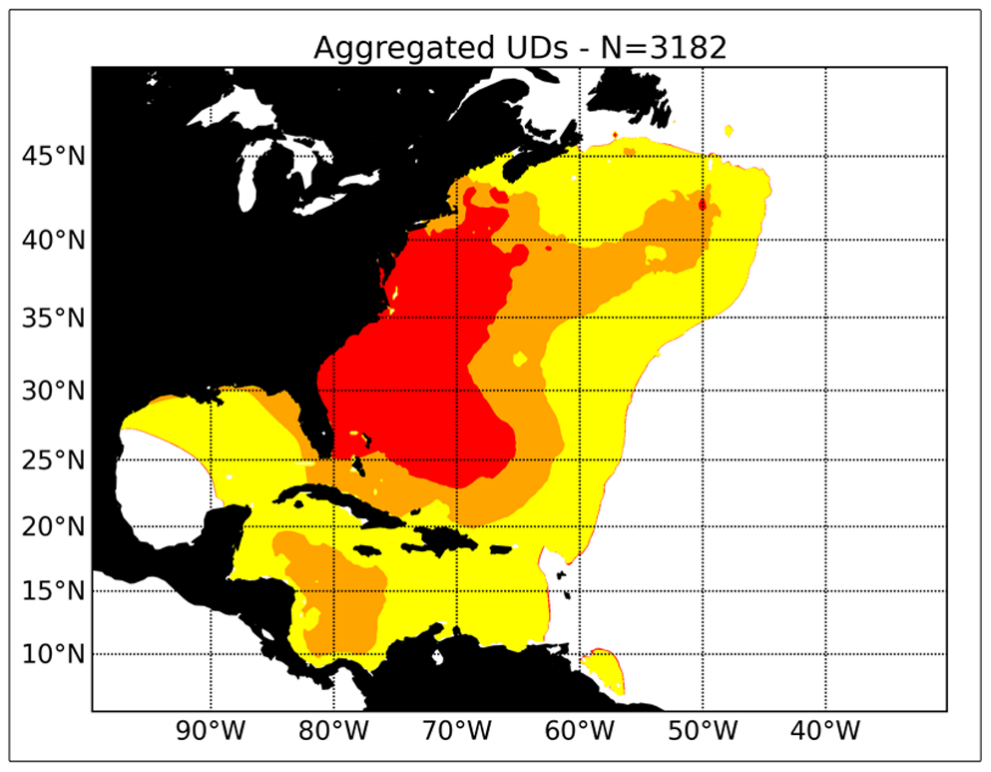
The overall distribution of immature swordfish (<179 cm) based on information from pop-up archival satellite results obtained by the National Marine Fisheries Service (NMFS), South Carolina Department of Natural Resources (DNR) and Fisheries and Oceans Canada (DFO). The contoured Utilization Distributions (UD) are normalized to one (100%), and the red areas represent normalized UD> 95%, the orange areas correspond with 95%> = UD>75%, the yellow areas are 75> = UD>50%, and the uncoloured areas are UD< =  50%. The same colour coding is employed in subsequent figures.

The overall distribution of immature swordfish is shown on Fig. 4. The centre of the distribution of such swordfish occurs relatively close to the eastern USA, south of Cape Cod to Florida and the Bahamas. The edges of the distribution include the Gulf of Mexico, the Caribbean Sea, and the northwest Atlantic east until about 50° W longitude.

**Figure 5:**
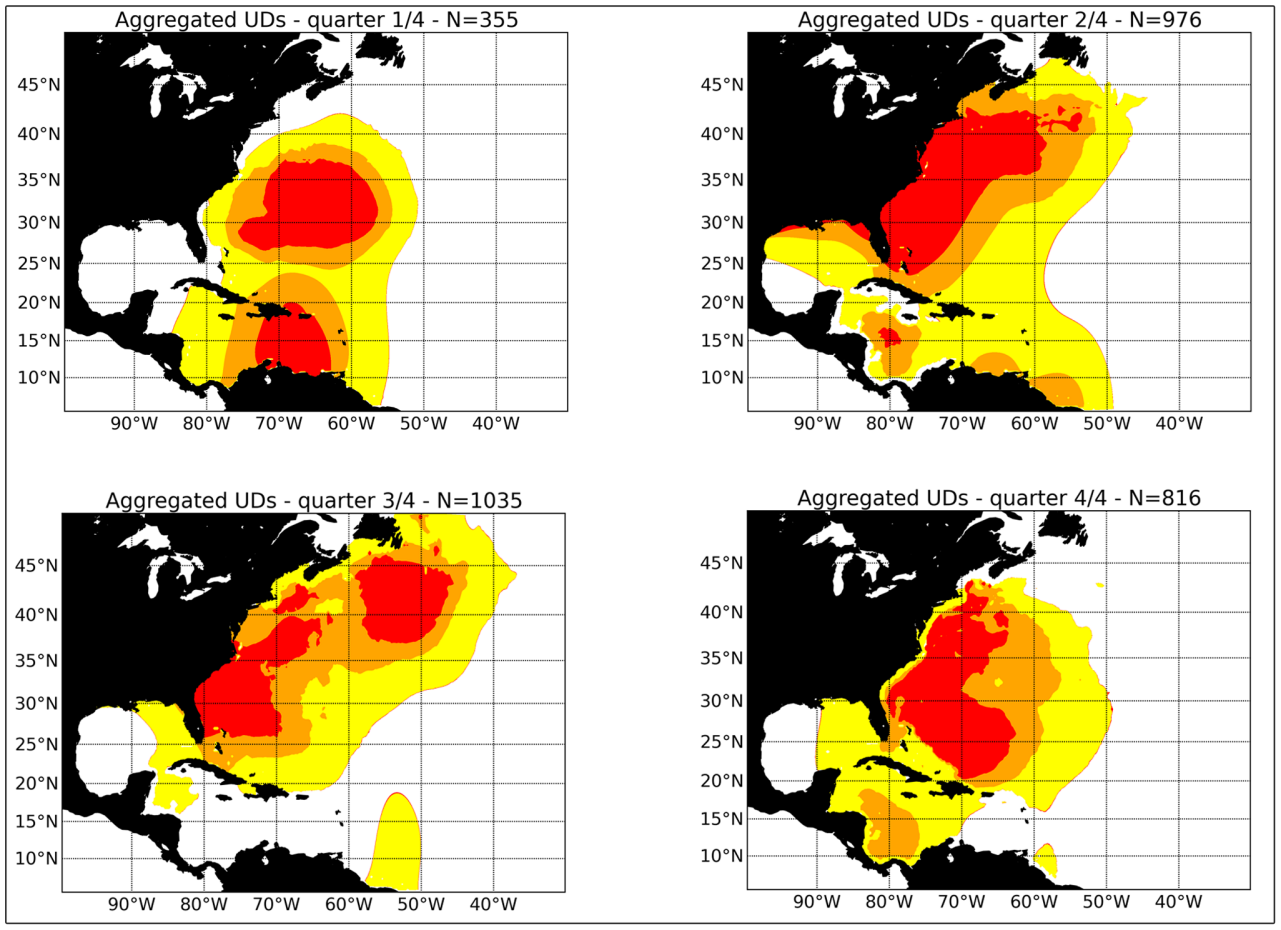
The seasonal distribution of immature swordfish (<179 cm) based on information from pop-up archival satellite results obtained by the National Marine Fisheries Service (NMFS), South Carolina Department of Natural Resources (DNR) and Fisheries and Oceans Canada (DFO).

The information in Fig. 4 is disaggregated into quarterly components in Fig. 5. The distribution of immature swordfish in Quarter One is centered in the Caribbean Sea region. In Quarter Two, the distribution has shifted northward, and is closely associated with coastal region of the eastern US, from southern Florida to Cape Cod. The northward movement continues in Quarter 3, and part of the distribution is now found further north and east, along the continent shelf edge off Nova Scotia and Newfoundland. The distribution of immature swordfish begins to retreat south in Quarter Four.

**Figure 6:**
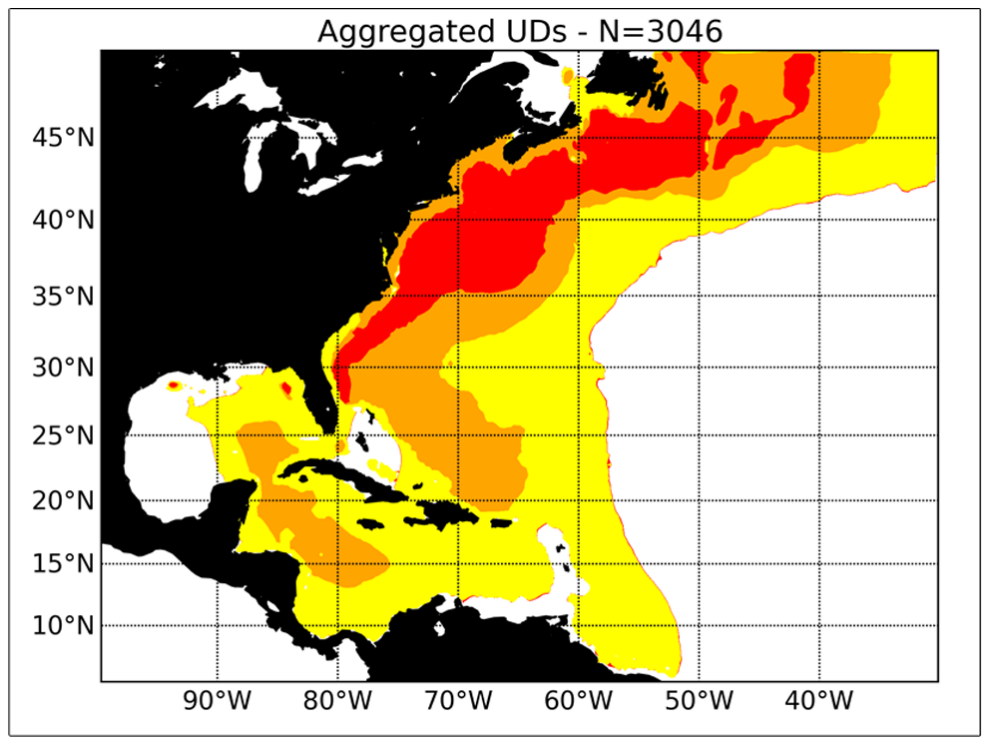
The overall distribution of mature swordfish ( = >179 cm) based on information from pop-up archival satellite results obtained by the National Marine Fisheries Service (NMFS), South Carolina Department of Natural Resources (DNR) and Fisheries and Oceans Canada (DFO).

Fig. 6 shows the overall distribution of mature swordfish as inferred from the PSAT information. The centre of the distribution extends through higher latitudes than was the case for the immature fish, with evidence for swordfish occurring in latitudes as high as 50° N. latitude. There are also indications that swordfish occur in the Gulf of Mexico, and the Caribbean Sea. Mature swordfish did not appear to move further south than about 10° N. latitude.

**Figure 7:**
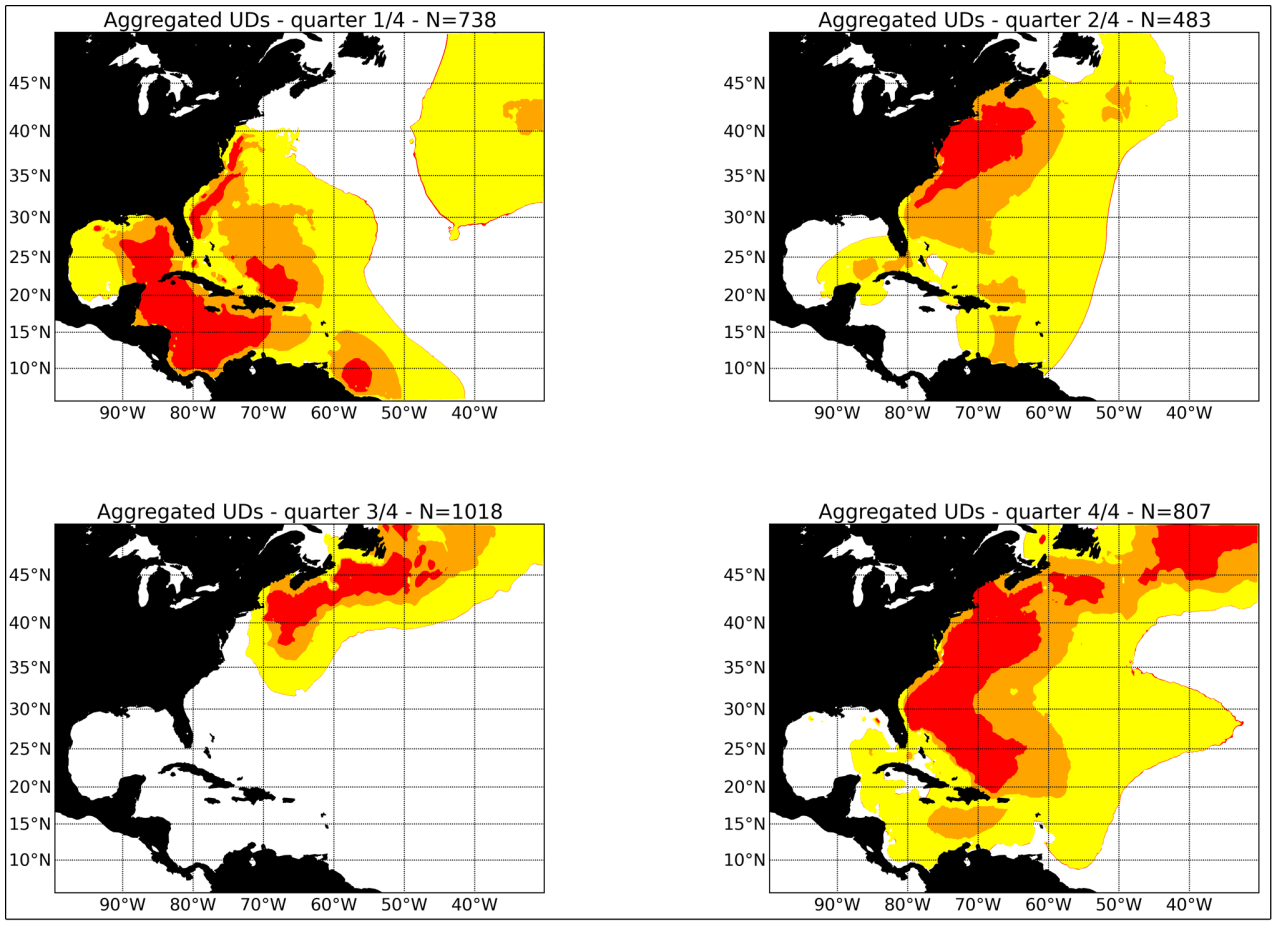
The seasonal distribution of mature swordfish ( = >179 cm) based on information from pop-up archival satellite results obtained by the National Marine Fisheries Service (NMFS), South Carolina Department of Natural Resources (DNR) and Fisheries and Oceans Canada (DFO).

The quarterly disaggregation of the information in Fig. 6 is presented in Fig. 7. In general, the larger mature swordfish show a more northerly distribution by quarter compared with the immature swordfish in our dataset. For example, in Quarter One, the centre of distribution extends as far north as about 40° N. latitude, compared with about 25° N latitude for the immature swordfish. By Quarter Two, the swordfish had moved north to 45° N latitude, and spread in an easterly direction around the Grand Banks of Newfoundland. By Quarter Three, the centre of distribution of the mature swordfish was in the Gulf of Maine, off Georges Bank, the Scotian Shelf and the Grand Banks of Newfoundland. By Quarter Four, the larger swordfish had begun their southward seasonal movement, with the southern margin of the distribution approaching the Caribbean Sea.

**Figure 8:**
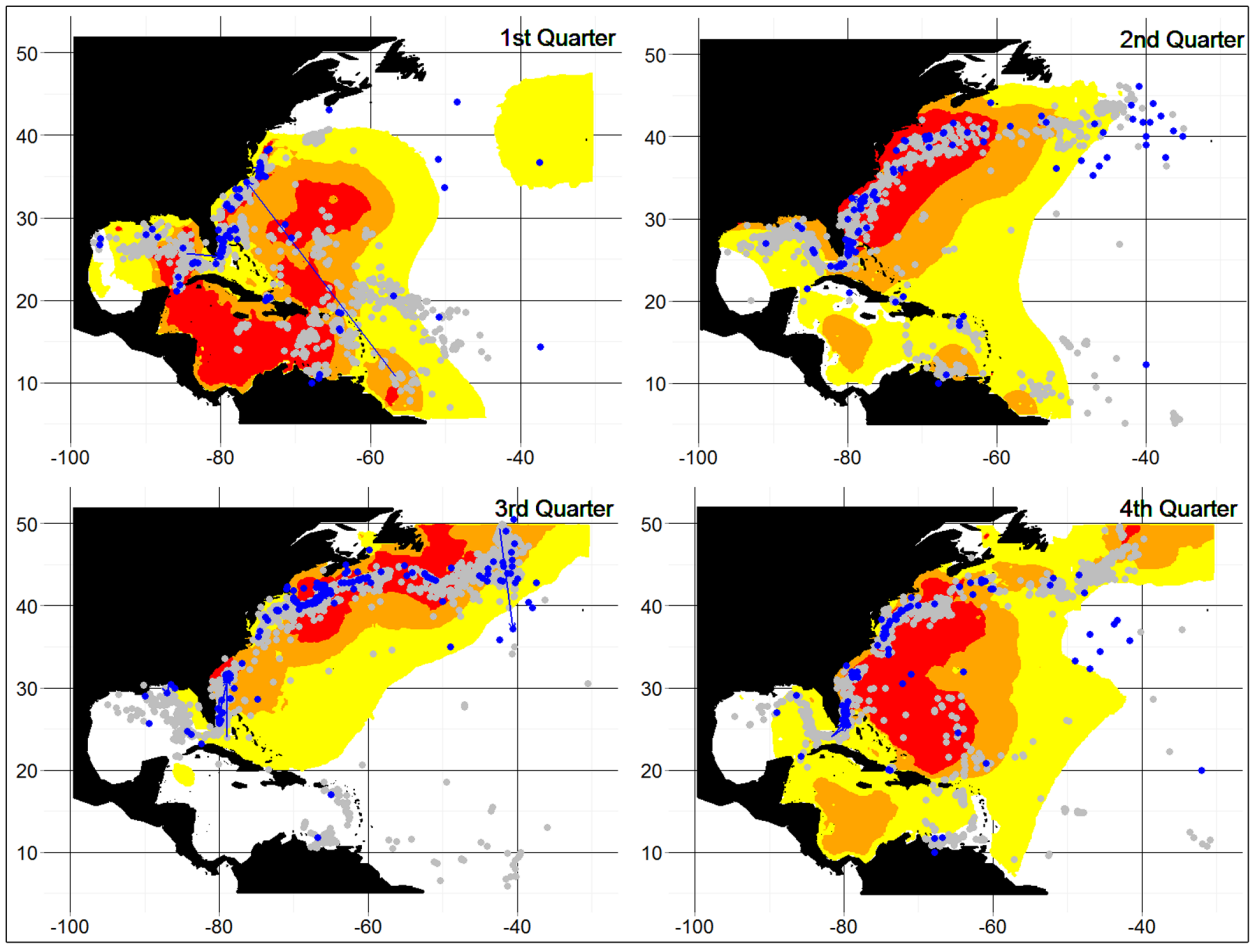
The seasonal distribution of both mature and immature swordfish obtained from the combined satellite archival tag data presented here, compared with conventional tagging data available from the International Commission for the Conservation of Atlantic Tunas, also shown by quarter. The release and recapture points for conventional tagging data are shown as grey and blue points, respectively. In the few cases where recaptures were made within the same quarter and year, an arrow is drawn between the release and recapture points.

An independent verification of our results is provided in Fig. 8, which compares the seasonal PSAT information on swordfish distributions for both immature and mature fish with the locations of swordfish conventional tagging events, including cases when a recapture was made. In general, the two indicators of swordfish distribution show good agreement. There were 2473, 2499, 4545 and 3583 records of tag recaptures and deployments in the conventional tagging database, for the first to fourth quarter respectively. Of those records, the proportion that fell within the swordfish distributions inferred from the PSAT information was 93.7, 95.2. 85.6 and 96.5%, for the first to fourth quarters respectively. In addition, areas of predicted high usage based on the PSAT data agreed well with aggregations of swordfish apparent from the conventional tagging studies (see, for examples Fig. 8 first quarter, eastern Gulf of Mexico and Atlantic coastal waters and third quarter, Gulf of Maine). However, there were also some instances where there were mismatches between the distributions inferred from PSAT data, and the observed distributions of conventional tagging deployments and recaptures (see for examples, Fig. 8 third quarter, eastern Gulf of Mexico and fourth quarter, coastal Venezuela.

## Discussion

Previous work on Atlantic swordfish estimated fish trajectories within the framework of Dynamic Generalized Linear Models [3]. The animal's locations were computed by maximizing a likelihood function taking into account SST constraints, the observations of sunrise and sunset times as well as the prior dynamics of the animal. The previous method is simpler, as the underlying distributions are assumed Gaussian (i.e. parameterized by a mean and a covariance) and are not constrained by coastline data.

In contrast, the HMM method implemented here provides a non-parametric probability density of the animal's position. HMM can take into account arbitrary observations models, non-Gaussian error sources and highly non-linear constraints such as shorelines or islands. This scheme is adapted to manipulate multi-modal distributions and should be favoured in data-poor scenarios, as was the case here. Although this method does not readily provide individual trajectories, the analyses of the generated UDs has provided important new information on the seasonal distribution of Atlantic swordfish in the northwest Atlantic.

By combining disparate data from three laboratories, this study has provided the most comprehensive examination of swordfish migration in the North Atlantic to date. There are several conclusions that have implications for the current view of swordfish stock structure and fisheries management. An earlier study has concluded that the seasonal movements exhibited by swordfish in the Northwest Atlantic appeared to be inconsistent with the management unit employed by ICCAT, which assumes a single stock unit throughout the North Atlantic [3]. The results showed no movements across the North Atlantic, and fish remained in the Northwest Atlantic, west of 50° W longitude. Our new findings, which include a considerably larger data set with a greater range of sizes and deployment locations (a recommendation made in [3]), generally support the conclusions of the earlier paper. We saw little evidence for movement of fish east of 40° W longitude (the greatest eastward movement shown by mature fish during Quarter Two (Fig. 7, see also the Supporting Information (Video S1, Video S2) illustrating the movement of two mature fish at liberty for more than 400 d). The Supporting Information also illustrates the previously noted tendency for swordfish to return to the same foraging areas on an annual basis [3].

While our data do not appear consistent with a population that is freely mixing in an east-west direction across the Atlantic, they are consistent with the convention for the boundary between north and south Atlantic stocks employed by ICCAT, which considers the division to be at 5° N. latitude. The exact location of the boundary is somewhat contentious, with some workers claiming that the division should be further north. For example, a genetic investigation of swordfish stock structure suggested that the boundary between the northern and southern stock may be located in the range of 10 to 20° N, and that individuals from the north and south populations may intermingle in the boundary zone [24]. Our results indicate that PSAT-tagged swordfish deployed in this study did not occur south of 5° N, thus supporting the current convention.

**Figure 9:**
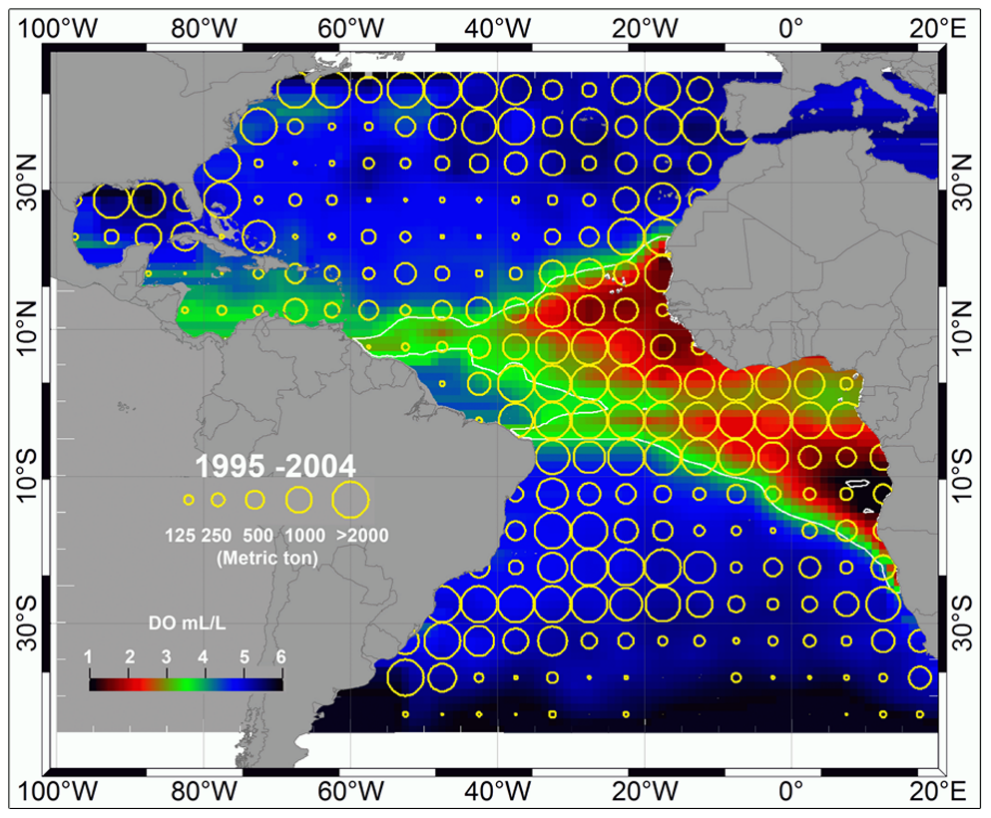
Atlantic Ocean-wide swordfish catches from the international longline fleets for the decade 1995–2004, shown in relation to dissolved oxygen concentration. Largest yellow circles are≥2000 mt. The catch data were provided by the International Commission for the Conservation of Atlantic Tunas.

In spite of the absence of movements of PSAT-tagged swordfish south of 5° N, there are, however, some data from other investigations that suggest that the management unit boundary could be reviewed. For instance, changes in the spatial distribution of the industrial longline fleets [25] and new genetic information [26] have prompted some to suggest that the current boundary could be revised. Swordfish are caught by longline fleets near the management boundary in substantial quantities (Figure 9), to the extent that there is no spatial discontinuity in the distribution of the catch, as would be expected if the management boundary delineated different stocks. However, this area is associated with the Oxygen Minimum Zone (OMZ), thus there is potential for hypoxia-based habitat compression resulting in a condensed distribution of predators, preferred prey, and fishing effort in these areas [27]. Under such circumstances, spatial discontinuities in catch distributions usually indicative of stock boundaries might not be expected. PSAT deployments on swordfish near the existing boundary could allow further evaluation of the suitability of the management boundary and also allow a better understanding of swordfish habitat use in the equatorial Atlantic and how it differs from swordfish in higher latitudes.

The greater scope of this study compared with [3] has allowed us to better describe seasonal habitat use of swordfish. For example, the swordfish released on Georges Bank described in [3] showed no affinity with the Gulf of Mexico, a known spawning area [28]. However, with the larger number of deployments in this study including a broader range of release sites, the distribution of mature fish during Quarter One (which includes the peak period of spawning, Fig. 7) is shown to include the Gulf of Mexico, the Caribbean Sea, and waters off eastern North America from 40° N. latitude south to about 20° N latitude. The areas of fish that were assumed to be mature in the first quarter (Fig. 7) generally concurs with the distribution of known spawning areas [28], but the PSAT results presented here show a broader distribution of potential spawners in the Caribbean Sea than was previously described [28].

We have used an independent data set (a database that contains the aggregated conventional tagging data provided by Contracting Parties to ICCAT) to validate the inferences made from the PSAT data, the first time such a comparative approach has been used in descriptions of fish distribution from electronic tagging studies, to our knowledge. The distribution of tagging releases and recaptures compared well with the information from PSATs, as >85% of locations of conventionally-tagged swordfish releases or recaptures were found within the Utilization Distribution corresponding with 75> = UD>50% (Fig. 8). However, there were some mismatches between the two datasets, noted earlier (for examples, see Fig. 8 third quarter, eastern Gulf of Mexico and fourth quarter, coastal Venezuela). Such apparent discrepancies, in part, could be due to the conventional tagging data being reliant on the distribution of fishing effort for recapture information, whereas the PSAT method is independent of the fishery. It could also reflect the fact that the data from the ICCAT conventional tagging database covered a considerably longer period (70 years, 1940 to 2009) than did the PSAT studies reported here (8 years, 2002 to 2009). There is evidence that swordfish occurred coastally during early periods of exploitation (see examples off eastern North America and South Africa, as shown in [28]), and in some cases, the coastal aggregations decreased markedly. Thus, during the 70-year period for the conventional tagging studies, the relative abundance of population components may have changed in comparison to their abundance during the period of PSAT studies.

## Supporting Information

Video S1 **The daily distributions of a PSAT-tagged individual swordfish released Aug. 5, 2008, Southern Grand Banks of Newfoundland, as inferred using the methods described in the current paper.** The fish was at liberty for 410 days.
(MPG)

Video S2 **The daily distributions of a PSAT-tagged individual swordfish released Aug. 5, 2008, Southern Grand Banks of Newfoundland, as inferred using the methods described in the current paper.** The fish was at liberty for 409 days.
(MPG)
