# Correction: Seasonal Distributions and Migrations of Northwest Atlantic Swordfish: Inferences from Integration of Pop-Up Satellite Archival Tagging Studies

**DOI:** 10.1371/journal.pone.0117961

**Published:** 2015-02-03

**Authors:** The PLOS ONE Staff

There is an error in the Competing Interests section. The number of authors that have affiliations with Datasio and Collecte Localisation Satellites should be four. The complete, correct competing interests information is as follows: While four authors have affiliations with the commercial companies Datasio and Collecte Localisation Satellites, this does not alter their adherence to PLOS ONE policies on sharing data and materials.

Additionally, there is an error in the final sentence of the Funding section. The last sentence should read: Data from the South Carolina Department of Natural Resources may be requested from Dr. Marcel Reichert (ReichertM@dnr.sc.gov).

Please view the complete, correct Funding section here: The authors confirm that, for approved reasons, some access restrictions apply to the data underlying the findings. Data are not owned by the authors of the paper but may be obtained from the relevant government agencies. Data from Fisheries and Oceans Canada may be requested from Dr. John D. Neilson (John.Neilson@dfo-mpo.gc.ca). Data from the US National Marine Fisheries Service may be requested from Dr. Clay Porch (Clay.porch@noaa.gov). Data from the South Carolina Department of Natural Resources may be requested from Dr. Marcel Reichert (ReichertM@dnr.sc.gov).
